# Aqueous−humor biomarkers and real−world efficacy of conbercept for diabetic macular edema: a prospective study in elderly patients

**DOI:** 10.3389/fendo.2025.1632878

**Published:** 2025-07-07

**Authors:** Min Xie

**Affiliations:** ^1^ Department of Ophthalmology, Luzhou Maternal and Child Health-Care Hospital, Luzhou, Sichuan, China; ^2^ Department of Ophthalmology, Luzhou Second People’s Hospital, Luzhou, Sichuan, China

**Keywords:** diabetic macular edema, conbercept, elderly, aqueous humor biomarkers, VEGF, IL-6

## Abstract

**Background:**

To evaluate real−world efficacy of conbercept and the predictive value of aqueous−humor biomarkers in elderly diabetic macular edema (DME).

**Methods:**

In this single−arm, prospective study, 150 patients ≥ 65 years received three monthly conbercept injections followed by pro−re−nata dosing over 6 months. Baseline vascular endothelial growth factor (VEGF) and interleukin−6 (IL−6) levels were quantified from aqueous humor; best−corrected visual acuity (BCVA) and central subfield thickness (CST) were monitored. Mixed−effects modelling and receiver−operating−characteristic (ROC) analysis explored associations between biomarkers and outcomes.

**Results:**

Mean BCVA improved by approximately nine ETDRS letters, and CST declined by about 85 µm at Month 6. Higher baseline VEGF and IL−6 were associated with greater CST reduction and moderately increased odds of a ≥ 15−letter gain; VEGF showed fair discriminative ability (AUC=0.74). Treatment was well−tolerated, with no unexpected ocular or systemic adverse events.

**Conclusion:**

Conbercept produced meaningful anatomical and functional benefits in an elderly DME cohort. Baseline aqueous VEGF and IL−6, while not definitive stand−alone tests, may help identify eyes likely to achieve pronounced anatomical improvement and warrant further investigation as components of a multi−marker predictive panel.

## Introduction

Diabetic Macular Edema (DME) is a significant complication of diabetes mellitus, particularly prevalent among the elderly, and is a leading cause of vision impairment globally. The prevalence of diabetes is increasing, with projections indicating it will affect 592 million individuals worldwide within the next two decades, thereby escalating the burden of DME (1). DME leads to fluid accumulation in the macula, resulting in vision loss and impaired daily functioning (2, 3). The pathophysiology of DME involves chronic hyperglycemia, which triggers microvascular damage and inflammatory pathways, leading to the breakdown of the blood-retinal barrier (BRB) and subsequent fluid leakage into the retinal layers (3, 4). Key mediators such as vascular endothelial growth factor (VEGF) and inflammatory cytokines play crucial roles in macular swelling (2, 5).

Current management of DME primarily involves anti-VEGF therapies, which have revolutionized treatment by effectively reducing macular thickness and improving visual acuity. Agents like ranibizumab and aflibercept are well-established in clinical practice (2, 6). Conbercept, another anti-VEGF agent, acts by binding multiple VEGF isoforms and placental growth factor (PlGF), showing promise in clinical trials, although specific data on its efficacy in the elderly population remains limited (6). Despite the effectiveness of these treatments, there are notable limitations, including inter-patient variability in response and the need for frequent injections (4, 7). This necessitates ongoing research into extended durability drugs and alternative therapeutic strategies to improve patient outcomes and reduce treatment burdens (5, 6).

The assessment of aqueous humor biomarkers in DME is crucial for understanding local disease activity and predicting treatment outcomes. Intraocular markers, such as cytokines in the aqueous humor, are significant because they directly reflect the local inflammatory and angiogenic processes occurring in the eye, unlike systemic markers which may not accurately represent ocular conditions (8, 9). Elevated levels of cytokines like VEGF, IL-6, and MCP-1 in the aqueous humor have been consistently associated with DME, indicating their potential as biomarkers for disease severity and treatment response (8, 9). Specifically, higher baseline levels of VEGF and IL-6 have been linked to less favorable anatomical and visual outcomes following anti-VEGF therapy (10, 11). Real-world studies are essential as they provide insights into the effectiveness of treatments in diverse patient populations (12, 13). Identifying factors that predict which elderly patients will benefit most from anti-VEGF therapy is crucial (13, 14). Therefore, real-world data can help tailor more effective and personalized treatment strategies, improving outcomes for patients with DME across various demographics (14, 15).

Despite the growing application of anti-VEGF therapies for DME, a critical gap remains in understanding conbercept’s real-world efficacy specifically among older patients, particularly with respect to intraocular biomarker profiles. Although elevated VEGF and IL−6 levels are consistently associated with greater baseline macular edema, published reports diverge on whether these biomarkers ultimately portend a favorable or unfavorable response to anti−VEGF treatment. Some studies indicate that eyes with higher VEGF experience larger CST reductions once angiogenic drive is suppressed, whereas persistent IL−6–mediated inflammation may limit visual recovery. The present study therefore prospectively evaluates how baseline VEGF and IL−6 together influence both anatomical and functional outcomes in an elderly DME population. By focusing on these older individuals, our findings could refine patient selection and guide more personalized conbercept regimens, ultimately improving both clinical outcomes and resource allocation.

## Methods

### Study design and setting

This was a single−arm, prospective, real-world, observational study conducted at Luzhou Second People’s Hospital, from August 2021 to March 2024, therefore, no randomization or parallel control group was employed. All patients received conbercept as part of routine care, and baseline values served as internal comparators for longitudinal analyses. The study adhered to the Declaration of Helsinki and received approval from the ethical committee of Luzhou Second People’s Hospital. Written informed consent was obtained from all participants prior to enrollment.

#### Inclusion criteria

1), Age ≥65 years, diagnosed with type 1 or type 2 diabetes mellitus; 2), Clinical and optical coherence tomography (OCT) confirmation of diabetic macular edema (DME) with a central subfield thickness (CST) ≥300 µm; 3), Best-corrected visual acuity (BCVA) between 20/40 and 20/400 (Snellen equivalent) or the corresponding Early Treatment Diabetic Retinopathy Study (ETDRS) letter score; 4), Willingness to provide written informed consent and comply with follow-up.

#### Exclusion criteria

1), Any ocular pathology other than DME significantly affecting BCVA (e.g., advanced glaucoma, vitreoretinal diseases not related to diabetes); 2), Prior intraocular surgery or laser treatment within 3 months before screening (except for uncomplicated cataract surgery >3 months earlier); 3), Active intraocular inflammation (e.g., uveitis) or uncontrolled intraocular pressure (IOP >25 mmHg despite medication); 4), Significant media opacities (e.g., dense cataract) preventing accurate OCT measurements; 5), Systemic conditions precluding compliance with study procedures (e.g., severe cardiac disease, inability to attend follow-up).

### Sample size estimation

A pilot chart review suggested an expected mean BCVA improvement of approximately +8 letters with a standard deviation (SD) of 5 letters after 6 months of anti-VEGF treatment in an elderly population. A minimum sample size of 120 was estimated to detect a 2-letter difference from historical controls with 80% power (α=0.05).

### Study procedures

A total of 150 patients (150 eyes) were enrolled. If both eyes satisfied eligibility criteria, the eye with poorer baseline BCVA was selected *a priori* as the study eye; the fellow eye received standard care but was not included in the analysis.

*Baseline Examination*: All participants underwent a comprehensive ophthalmic examination, including slit-lamp biomicroscopy, dilated fundus examination, tonometry for IOP measurement, and BCVA assessment using ETDRS charts at 4 meters. Spectral-domain OCT (SD-OCT) was performed to measure CST using the Spectralis HRA+OCT platform (Heidelberg Engineering, Heidelberg, Germany; software version 6.16). A standardized 20° × 20° macular volume scan (49 B−scans; 512 A−scans per line; automatic real−time averaging = 9) was acquired for each eye at baseline and follow−up visits. CST was automatically calculated by the instrument’s built−in segmentation algorithm as the mean retinal thickness within the central 1−mm ETDRS grid zone; all scans were reviewed by two masked graders, and segmentation errors were manually corrected when necessary.

Additional data, including demographics (age, sex), medical history (duration of diabetes, comorbidities), and systemic parameters (HbA1c, blood pressure), were recorded.

### Aqueous humor collection and biomarker assay

Aqueous humor (AH) was collected immediately prior to the first intravitreal injection of conbercept. A subset of patients who required additional procedures or injections at Month 3 or Month 6 also underwent optional repeat sampling, if clinically indicated and approved by the ethics committee. Under topical anesthesia and aseptic conditions, a 0.1 mL aliquot of AH was aspirated via a 30-gauge needle inserted at the peripheral cornea, ensuring minimal risk to patient safety. Samples were transferred into sterile, RNase/DNase-free Eppendorf tubes and immediately stored at -80°C for later batch analysis.

VEGF, IL-6, IL-8, TNF-α, and bFGF were measured using a multiplex bead-based immunoassay following the manufacturer’s protocol. The lower limit of detection for each analyte was documented; samples below that threshold were assigned a nominal value (e.g., half the limit).

### Conbercept injection protocol

#### Loading phase (months 0–2)

Three monthly intravitreal injections of 0.5 mg conbercept (Chengdu Kanghong Biotech, China) were administered in the study eye(s).

#### Maintenance phase (months 3–6)

Participants received intravitreal conbercept pro re nata (PRN) based on retinal thickness (CST ≥300 µm or new/substantial fluid on OCT) and/or persistent visual impairment attributable to DME.

#### Injection technique

Standard aseptic procedure was used. Topical anesthesia was achieved with 0.5% proparacaine hydrochloride ophthalmic solution (Alcaine^®^, Alcon, Fort Worth, TX, USA); one drop was instilled three times at one−minute intervals during the five minutes preceding both aqueous humor sampling and intravitreal injection. Immediately afterward, 5% povidone−iodine was applied to the conjunctival sac and periocular skin in keeping with standard aseptic protocol. After topical anesthesia, a 30-gauge needle was inserted 3.5–4.0 mm posterior to the limbus in the superotemporal quadrant. Post-injection, IOP was measured within 30 minutes, and topical antibiotics were prescribed for 3 days.

After the initial three monthly conbercept injections, patients were evaluated at each visit for signs of persistent or recurrent macular edema. In this real-world setting, re-treatment was guided primarily by 1), Recurrence of intraretinal or subretinal fluid, or a central subfield thickness (CST) ≥300 µm compared with prior scans; 2), A decline of ≥5 ETDRS letters from the previous best-measured BCVA; 3), In cases where the above thresholds were borderline, clinician judgment factored in patient symptoms, comorbidities, and overall tolerance of injections.

### Statistical analysis

All statistical analyses were performed using R (version 4.4), with continuous data presented as mean ± standard deviation or median [interquartile range] and categorical data as frequencies (%). Between-group comparisons (e.g., mild vs. moderate/severe DME) were made using the independent samples t-test or Mann-Whitney U test for continuous variables and the chi-square or Fisher’s exact test for categorical variables, while within-group changes over time (e.g., baseline vs. Month 6) were evaluated with paired t-tests or Wilcoxon signed-rank tests. Where repeated measurements were involved (e.g., monthly BCVA assessments), repeated-measures ANOVA or linear mixed-effects modeling was used to account for both fixed (time, biomarker levels) and random (inter-individual) effects. Correlations between continuous variables were calculated using Pearson correlation coefficients, and receiver operating characteristic (ROC) curve analyses were conducted to assess the discriminative power of aqueous biomarkers in predicting treatment response. Logistic regression was applied to identify independent predictors of a dichotomous outcome (≥15-letter gain), and all significant variables from univariable analysis were entered into a multivariable model. Unless otherwise indicated, statistical significance was set at a two-tailed p-value <0.05, and where appropriate, multiple comparison adjustments (e.g., Bonferroni) were utilized to control Type I error rates.

## Results

A total of 150 elderly patients with diabetic macular edema (DME) were enrolled. As shown in **Table 1**, the mean age was 73.4 ± 5.2 years (range, 65–89), and 50.7% were male. The average duration of diabetes was 12.8 ± 5.6 years, with a mean HbA1c of 7.9 ± 1.2%. Most participants (62.7%) were phakic, and 62.7% had coexisting hypertension. Baseline best-corrected visual acuity (BCVA) was 50.3 ± 8.2 letters, and the central subfield thickness (CST) was 445.6 ± 65.3 µm.

**Table 1:** Baseline demographics and clinical characteristics.

Characteristic	Value
Age (years), mean ± SD	73.4 ± 5.2 (range 65–89)
Gender, n (%)	
Male	76 (50.7%)
Female	74 (49.3%)
Duration of Diabetes (years), mean ± SD	12.8 ± 5.6
HbA1c, % ± SD	7.9 ± 1.2
Hypertension, n (%)	94 (62.7%)
Hyperlipidemia, n (%)	71 (47.3%)
Lens Status, n (%)	
Phakic	94 (62.7%)
Pseudophakic	56 (37.3%)
Baseline BCVA, mean ± SD	50.3 ± 8.2
Baseline CST (µm), mean ± SD	445.6 ± 65.3
IOP (mmHg), mean ± SD	15.2 ± 2.1
Previous Anti-VEGF Treatment, n (%)	19 (12.7%)
Previous Focal Laser, n (%)	32 (21.3%)

BCVA, best-corrected visual acuity; CST, central subfield thickness; IOP, intraocular pressure.

Data are presented as mean ± standard deviation or n (%).

Prior to the first conbercept injection, aqueous humor samples were collected and analyzed for VEGF, IL-6, IL-8, TNF-α, and bFGF. **Table 2** summarizes these baseline biomarker levels, with mean ± SD values of 90.5 ± 35.2 pg/mL for VEGF and 45.8 ± 18.1 pg/mL for IL-6. The baseline levels of IL-8, TNF-α, and bFGF were 30.3 pg/mL, 8.7 pg/mL and 18.9 pg/mL, respectively.

**Table 2:** Baseline aqueous humor biomarker levels.

Biomarker	Mean ± SD	Median [IQR]	Range
VEGF (pg/mL)	90.5 ± 35.2	85.0 [60.2–110.6]	25–180
IL-6 (pg/mL)	45.8 ± 18.1	42.0 [30.8–59.5]	10–95
IL-8 (pg/mL)	30.3 ± 12.4	29.1 [18.6–40.5]	8–65
TNF-α (pg/mL)	8.7 ± 3.1	8.2 [6.0–11.0]	2–17
bFGF (pg/mL)	18.9 ± 7.5	18.0 [12.1–24.5]	5–35

VEGF, vascular endothelial growth factor; IL, interleukin; TNF-α, tumor necrosis factor-alpha; bFGF, basic fibroblast growth factor.

All participants received a three-injection loading phase over the first 3 months. Subsequent treatments were administered pro re nata (PRN). As detailed in **Table 3**, the mean number of injections at 6 months was 4.2 ± 1.1, with 62 patients (41.3%) requiring one additional post-loading injection, and 21.3% needing two or more. Adjunct focal laser was used in 7.3% of cases, mainly for persistent or focal edema.

**Table 3:** Injection frequency and additional interventions over 6-month follow-up.

Variable	Value
Mean Number of Conbercept Injections	4.2 ± 1.1
Patients Receiving 3-Load Injections, n (%)	150 (100.0%)
PRN Injections After Loading, n (%)	
1 additional injection	62 (41.3%)
≥2 additional injections	32 (21.3%)
Adjunct Focal Laser, n (% of total)	11 (7.3%)
New Systemic Therapy Changes, n (%)	
Glycemic control intensification	29 (19.3%)
Blood pressure medication adjustment	38 (25.3%)

All patients underwent the standard 3-month loading phase. “PRN” indicates pro re nata (as needed) treatment based on OCT and clinical findings.

Marked improvements in BCVA and CST were observed. At 6 months, BCVA increased by +8.6 ± 4.6 letters from baseline (p<0.001) and CST decreased by -85.1 ± 40.2 µm (p<0.001) (**Table 4**). Approximately 30.7% of eyes achieved a ≥10-letter gain, and 20.7% reached a ≥15-letter improvement.

**Table 4:** Changes in BCVA and CST over time.

Time Point	BCVA	Δ BCVA from Baseline	CST (µm)	Δ CST from Baseline
Baseline	50.3 ± 8.2	—	445.6 ± 65.3	—
Month 1	54.8 ± 9.1	+4.5 ± 3.2*	400.2 ± 58.7	-45.4 ± 28.2*
Month 3	57.6 ± 8.7	+7.3 ± 4.8*	373.2 ± 55.1	-72.4 ± 36.5*
Month 6	58.9 ± 9.0	+8.6 ± 4.6*	360.5 ± 51.2	-85.1 ± 40.2*

Data shown as mean ± SD. *P < 0.001 vs. baseline (paired t-tests or repeated-measures ANOVA).

BCVA, best-corrected visual acuity; CST, central subfield thickness; Δ, change from baseline.

Baseline VEGF remained the only cytokine that retained statistical significance across all anatomical metrics after multiple−comparison correction: it correlated positively with absolute CST change (r = +0.33, FDR−adjusted p = 0.020) and, importantly, with percentage CST reduction (r = +0.31, FDR−adjusted p = 0.025) (**Table 5**). By contrast, IL−6 lost significance when baseline thickness was normalized (r = +0.15, FDR−adjusted p = 0.220), suggesting its previously observed association was largely driven by greater initial oedema rather than intrinsic sensitivity to therapy. Neither IL−8, TNF−α, nor bFGF showed significant relations with anatomical outcomes once FDR correction was applied (**Table 5**). Functionally, higher VEGF and IL−6 levels remained negatively correlated with BCVA gain at the unadjusted level (r = –0.37 and –0.26, respectively), but only VEGF showed an FDR−adjusted p−value below 0.05 (0.020) (**Table 5**). Collectively, these findings indicate that VEGF provides independent, baseline−adjusted predictive information for anatomical response, whereas IL−6 primarily reflects disease severity rather than superior therapeutic efficacy.

**Table 5:** Correlation of biomarker levels and changes in BCVA/CST at month 6.

Biomarkers	r (BCVA Change)	p-value (BCVA)	FDR p-value (BCVA)	r (CST Change)	p-value (CST)	FDR p-value (CST)	r (% CST reduction)	p-value	FDR p value
VEGF	-0.37	0.002	0.020	+0.33	0.004	0.020	+0.31	0.005	0.025
IL-6	-0.26	0.018	0.060	+0.22	0.032	0.080	+0.15	0.110	0.220
IL-8	-0.21	0.045	0.090	+0.18	0.071	0.101	+0.11	0.188	0.250
TNF-α	-0.19	0.059	0.098	+0.14	0.096	0.120	+0.08	0.276	0.304
bFGF	-0.10	0.225	0.250	+0.09	0.280	0.280	+0.04	0.551	0.551

Pearson (r) correlation coefficients are shown.

Serious ocular adverse events were rare (1 case of endophthalmitis, 0.7%), and IOP elevations requiring medication occurred in 5.3% of patients (**Table 6**). Mild anterior chamber inflammation, subconjunctival hemorrhage, and cataract progression were also documented but did not necessitate study discontinuation in most cases.

**Table 6:** Summary of adverse events over 6 months.

Adverse Event	Number of Events	Patients Affected, n (%)
IOP Elevation Requiring Medication	9	8 (5.3%)
Mild Anterior Chamber Inflammation	6	6 (4.0%)
Subconjunctival Hemorrhage	4	4 (2.7%)
Cataract Progression (Phakic only)	3	3 (3.2% of phakic eyes)
Endophthalmitis	1	1 (0.7%)
Systemic Events	2 (1 stroke, 1 MI)	2 (1.3%)
Total Serious Ocular Events	1	1 (0.7%)

Participants were stratified into mild vs. moderate/severe DME based on baseline CST (<400 vs. ≥400 µm). Patients with moderate/severe DME had a higher baseline CST (470.5 ± 55.2 vs. 373.6 ± 21.4 µm, p<0.001), required more injections (4.5 ± 1.1 vs. 3.8 ± 1.2, p=0.005), and exhibited a slightly larger CST reduction (–86.2 ± 36.7 vs. –61.2 ± 28.5 µm, p=0.001) (**Table 7**). Despite these differences, final BCVA at 6 months was comparable across subgroups (**Table 7**).

**Table 7:** Subgroup analysis of efficacy and biomarker levels by baseline DME severity.

Parameter	Mild DME (n=62)	Moderate/Severe DME (n=88)	p-value
Baseline CST (µm)	373.6 ± 21.4	470.5 ± 55.2	<0.001
Month 6 CST (µm)	312.4 ± 25.6	384.3 ± 42.1	<0.001
Δ CST (µm)	-61.2 ± 28.5	-86.2 ± 36.7	0.001
Baseline BCVA (letters)	52.1 ± 7.6	49.2 ± 8.4	0.012
Month 6 BCVA (letters)	59.5 ± 8.3	58.4 ± 9.2	0.384 (ns)
Δ BCVA (letters)	+7.4 ± 4.1	+9.2 ± 4.8	0.030
Baseline VEGF (pg/mL)	78.4 ± 31.0	98.6 ± 37.2	0.002
Baseline IL-6 (pg/mL)	40.1 ± 16.7	49.6 ± 18.2	0.001
Number of Injections (6 mos)	3.8 ± 1.2	4.5 ± 1.1	0.005
Good Responders (≥15 letters gained), n	8 (12.9%)	23 (26.1%)	0.046

A logistic model was employed to identify factors predicting a robust visual improvement (≥15 letters gained). Baseline CST (OR=1.08 per 10 µm, p=0.008), VEGF (OR=1.11 per 10 pg/mL, p<0.001), and IL-6 (OR=1.16 per 5 pg/mL, p=0.014) emerged as independent predictors (**Table 8**). Conversely, neither age nor diabetes duration significantly influenced the odds of a ≥15-letter gain.

**Table 8:** Multivariable logistic regression for predicting good visual response at month 6.

Variable	OR (95 % CI)	p-value
Age (per 1-year)	0.97 (0.93–1.02)	0.28
Baseline BCVA (letters)	0.95 (0.90–1.00)	0.05
Baseline CST (per 10 µm)	1.08 (1.02–1.14)	0.01
VEGF (per 10 pg/mL)	1.10 (1.04–1.17)	<0.001
IL-6 (per 5 pg/mL)	1.15 (1.02–1.30)	0.02
HbA1c (per 1%)	0.98 (0.87–1.11)	0.75
Diabetes duration (years)	1.02 (0.98–1.05)	0.36
Constant	—	0.023

VEGF displayed the highest area under the curve (AUC=0.74, p<0.001). IL-6 had an AUC of 0.68, whereas IL-8, TNF-α, and bFGF demonstrated comparatively weaker predictive capabilities (**Table 9**).

**Table 9:** ROC curve analysis for baseline biomarkers in predicting good visual Response.

Biomarker	AUC	95% CI	Sensitivity (%)	Specificity (%)	p-value
VEGF	0.74	0.66–0.82	76.5	69.3	<0.001
IL-6	0.68	0.60–0.77	70.6	62.4	0.002
IL-8	0.61	0.51–0.70	58.8	63.2	0.074
TNF-α	0.58	0.48–0.68	54.0	60.0	0.139
bFGF	0.54	0.44–0.64	52.9	57.3	0.387

A linear mixed-effects model incorporating multiple time points (baseline, 1, 3, 6 months) confirmed a significant average monthly improvement in BCVA (+1.26 letters/month, p<0.001). Patients with “high VEGF” (>80 pg/mL) started with a lower initial BCVA but gained letters faster over time, reflected in a positive time × VEGF interaction (p=0.031) (**Table 10**).

**Table 10:** Repeated measures analysis of BCVA over time using a linear mixed-effects model.

Fixed Effect	Estimate (SE)	95% CI	p-value
Intercept (Baseline)	50.2 (1.2)	47.9 - 52.5	<0.001
Time (per month)	1.26 (0.15)	0.96 - 1.57	<0.001
High VEGF Group (Yes=1)	-2.70 (1.25)	-5.16 - -0.24	0.032
Time × High VEGF Group	0.55 (0.25)	0.05 - 1.05	0.031
Age (per year)	-0.06 (0.03)	-0.12 - 0.00	0.059
Random Effects (Patient)	—	—	—

## Discussion

Our findings indicate that conbercept therapy produced marked anatomical (CST) and functional (BCVA) improvements in this elderly DME cohort. These gains occurred alongside biomarker patterns—higher baseline VEGF and IL−6 linked to larger CST reductions—that refine our understanding of treatment response. Our findings indicated a notable improvement in BCVA and a significant reduction in CST over six months of conbercept therapy, aligning with results from both randomized trials and observational studies where BCVA improvements of 0.41 ± 0.39 to 0.23 ± 0.20 logMAR and considerable CMT reductions have been documented (16, 17). Although one major trial did not observe a statistically significant CMT decrease (p = 0.385) (16), other long-term observational reports have shown meaningful reductions (e.g., from 510.9 ± 186.1 µm to 277.1 ± 122.9 µm) alongside a favorable safety profile and no severe adverse events (17). Our average injection frequency during the study period was lower than the 10.6 ± 2.0 injections reported over two years in a broader DME cohort (17), possibly reflecting differences in treatment protocols or comorbidity burdens in our older population. Regarding aqueous humor biomarkers, our correlation patterns for VEGF and IL-6 largely concur with prior studies indicating that these cytokines correlate with disease activity and thickness measures (18, 19), though variations in assay methods, sample sizes, and patient demographics—such as those with advanced non-proliferative diabetic retinopathy or prior panretinal photocoagulation—can lead to heterogeneous results (9, 20–22). Furthermore, our finding that older patients can exhibit elevated baseline cytokine levels is consistent with studies attributing age-related rises to both systemic and ocular factors (8, 21, 23). When CST reduction was expressed as a percentage of baseline thickness and baseline CST was entered as a covariate, VEGF—but not IL−6—remained an independent predictor, suggesting that VEGF carries predictive information beyond initial edema burden, whereas IL−6 predominantly reflects baseline severity. This distinction reconciles the paradox of robust anatomical response yet modest functional gain. Although baseline VEGF yielded the highest discriminative value in our cohort (AUC = 0.74), this level of accuracy is considered moderate. Accordingly, VEGF alone is unlikely to serve as a definitive gatekeeper for treatment intensification. Previous DME studies have reported similar single−marker AUCs (≈0.65–0.78), underscoring the inherent complexity of predicting therapeutic response from one molecule. Future work should evaluate combined models—including VEGF, IL−6, and structural OCT parameters—that may achieve higher composite AUCs and more clinically actionable thresholds.

Mechanistically, our data support the concept that elevated VEGF and IL-6 drive both angiogenic and inflammatory pathways, respectively, which can yield strong anatomical responses while producing variable visual outcomes (24–27). It is plausible that VEGF blockade successfully mitigates neovascular leakage and edema, but persistent inflammation modulated by IL-6 may limit functional gains, a notion consistent with reports describing robust anatomical improvements yet incomplete visual recovery (28, 29). Age-related physiological changes and systemic comorbidities may alter pharmacokinetics or ocular tissue susceptibility, potentially leading to delayed or atypical responses relative to younger cohorts (28, 29). This underscores the multifactorial nature of DME pathophysiology and the importance of integrating biomarker data into individualized treatment regimens, especially as age, metabolic status, and inflammatory burden converge to shape outcomes in this heterogeneous population.

Advanced age significantly impacts pharmacodynamics and clinical responses, primarily due to physiological changes that alter drug efficacy and safety. As individuals age, there is a decline in organ function, leading to decreased renal and hepatic clearance, which affects drug metabolism and elimination (30, 31). This results in an increased volume of distribution for lipid-soluble drugs and prolonged half-lives (30). Furthermore, older adults often exhibit heightened sensitivity to certain medications, particularly those affecting the central nervous system, such as benzodiazepines and opioids, due to age-related declines in CNS function (31, 32). Cardiovascular pharmacodynamics also show variability, with some agents demonstrating reduced effectiveness, while others may have increased effects in treatment-naive elderly patients (32). The complexity of managing medications in this population is compounded by polypharmacy and the potential for drug-drug interactions, particularly with direct oral anticoagulants used for venous thromboembolism (33). Overall, understanding these age-related changes is crucial for optimizing pharmacotherapy in geriatric patients (34).

The six-month follow-up may be insufficient for detecting long-term efficacy or potential late-onset complications, and the single-center setting could introduce selection bias or limit the applicability of our conclusions. Another limitation is the absence of randomization and a formal control group. Although this design enhances external validity by reflecting everyday practice, it precludes direct causal inference relative to untreated or alternative−treatment cohorts. We partially mitigated this limitation by adjusting for baseline covariates in multivariable mixed−effects models. We also acknowledge that biomarker levels can fluctuate based on subclinical inflammation, sample-handling variations, or collection timing, making consistent measurement crucial for robust analysis. Aqueous VEGF and IL−6 measured only at baseline cannot reflect the changes before and after treatment, serial sampling could have clarified whether conbercept directly modulates these cytokines over time or whether reductions correlate with anatomical response. Ethical and practical constraints precluded additional paracenteses in this elderly cohort, but future studies incorporating micro−volume sampling or tear−film surrogates are warranted. Although we employed strategies such as electronic record checks and phone reminders to minimize dropouts, missing data could still affect the generalizability of the results. Clinically, baseline cytokine levels may guide patient selection and individualized anti-VEGF regimens—particularly in those with elevated VEGF or IL-6 who might require more frequent injections or adjunctive anti-inflammatory agents—and future work should consider longer follow-up, comparative trials against other agents or implants, and biomarker-guided thresholds for therapeutic intensification. Further molecular and genetic studies could refine this biomarker-driven model, ultimately supporting more personalized and durable treatment strategies for elderly patients with DME.

In conclusion, our findings underscore the strong efficacy of conbercept in improving visual and anatomical outcomes among older diabetic macular edema patients, while also highlighting the pivotal role that baseline aqueous humor biomarker levels—particularly VEGF and IL-6—can play in identifying those most likely to benefit. These results collectively emphasize that incorporating biomarker profiling into routine assessment could enhance therapeutic decision-making, enabling clinicians to optimize treatment frequencies and potentially integrate adjunct anti-inflammatory strategies.

## Data Availability

The raw data supporting the conclusions of this article will be made available by the authors, without undue reservation.
